# Altered Intra- and Inter-Network Connectivity in Drug-Naïve Patients With Early Parkinson’s Disease

**DOI:** 10.3389/fnagi.2022.783634

**Published:** 2022-02-14

**Authors:** Weiqi Zeng, Wenliang Fan, Xiangchuang Kong, Xiaoming Liu, Ling Liu, Ziqin Cao, Xiaoqian Zhang, Xiaoman Yang, Chi Cheng, Yi Wu, Yu Xu, Xuebing Cao, Yan Xu

**Affiliations:** ^1^Department of Neurology, Union Hospital, Tongji Medical College, Huazhong University of Science and Technology, Wuhan, China; ^2^Department of Radiology, Union Hospital, Tongji Medical College, Huazhong University of Science and Technology, Wuhan, China; ^3^Hubei Province Key Laboratory of Molecular Imaging, Wuhan, China; ^4^Department of Chemistry, Emory University, Atlanta, GA, United States

**Keywords:** Parkinson’s disease, drug-naïve, functional MRI (fMRI), non-motor symptoms, resting-state functional connectivity

## Abstract

The aim of our study was to investigate differences in whole brain connectivity at different levels between drug-naïve individuals with early Parkinson’s disease (PD) and healthy controls (HCs). Resting-state functional magnetic resonance imaging data were collected from 47 patients with early-stage, drug-naïve PD and 50 HCs. Functional brain connectivity was analyzed at the integrity, network, and edge levels; UPDRS-III, MMSE, MOCA, HAMA, and HAMD scores, reflecting the symptoms of PD, were collected for further regression analysis. Compared with age-matched HCs, reduced functional connectivity were mainly observed in the visual (VSN), somatomotor (SMN), limbic (LBN), and deep gray matter networks (DGN) at integrity level [*p* < 0.05, false discovery rate (FDR) corrected]. Intra-network analysis indicated decreased functional connectivity in DGN, SMN, LBN, and ventral attention networks (VAN). Inter-network analysis indicated reduced functional connectivity in nine pairs of resting-state networks. At the edge level, the LBN was the center of abnormal functional connectivity (*p* < 0.05, FDR corrected). MOCA score was associated with the intra-network functional connectivity strength (FC) of the DGN, and inter-network FC of the DGN-VAN. HAMA and HAMD scores were associated with the FC of the SMN and DGN, and either the LBN or VAN, respectively. We demonstrated variations in whole brain connections of drug-naïve patients with early PD. Major changes involved the SMN, DGN, LBN, and VSN, which may be relevant to symptoms of early PD. Additionally, our results support PD as a disconnection syndrome.

## Introduction

Parkinson’s disease (PD) is a progressive neurodegenerative disorder, described as the loss of dopaminergic neurons in the substantia nigra pars compacta. Classic motor symptoms manifest as tremors, rigidity, and bradykinesia, which can be alleviated by dopaminergic replacement therapy. Parkinson’s disease pathology—characterized by the deposition of α-synuclein and formation of Lewy bodies—has confirmed that brain lesions are silently activated prior to the onset of clinical symptoms (Braak et al., 2004; Schapira et al., 2017).

Previous studies on resting state functional magnetic resonance imaging (rs-fMRI) reported that functional connectivity in the frontoparietal regions (de Schipper et al., 2018), occipital cortex (Canu et al., 2015), temporal gyrus (Chen et al., 2015), striatum (Hacker et al., 2012; Agosta et al., 2014), thalamus (Mueller et al., 2019), and basal ganglia (Ma et al., 2017) are affected in PD; thus, PD has been proposed to be a disconnection syndrome owing to its widely affected brain regions and complicated clinical symptoms (Cronin-Golomb, 2010). A previous study showed changes in whole-brain connectivity among drug-naïve patients using a topological analysis (De Micco et al., 2021a). Another study revealed dynamic changes in the sensorimotor and top-down control networks in patients with PD before and after medication (Chen et al., 2021).

However, current research on rs-fMRI for PD mainly focuses on patients with advanced PD and explores the association between specific symptoms, such as motor symptoms or cognitive impairment, and brain connectivity in specific regions (Li et al., 2019; Chung et al., 2020; Jung et al., 2020); the changes in intra- and inter-network brain connectivity in early PD remain unknown. Additionally, chronic levodopa treatment may significantly alter the brain connectivity network of patients, and some abnormal brain connectivity, which is not conducive to the study of pathological brain connections, may be corrected (Yang et al., 2016b; Day et al., 2019; Mueller et al., 2019). Thus, this study aimed to investigate the changes in whole-brain connectivity at three different levels in early PD patients who have never received dopaminergic therapy. We hypothesized that the intra- and inter-network brain connectivity may reveal a specific pattern of changes in drug-naïve individuals with early-stage PD that correlates with a series of clinical manifestations. Our findings may aid in our understanding of the mechanisms underlying PD development, as well as reveal the essence of the disease.

## Materials and Methods

### Participants and Clinical Assessment

Drug-naïve patients with PD (*n* = 56) were recruited from the Department of Neurology, Wuhan Union Hospital, and 54 age-matched healthy controls (HCs) were recruited from Physical Examination Center between June 2018 and December 2020 (Supplementary Figure 1). An X banner advertisement in the Outpatient Department was made to recruit participants. All participants were evaluated by experienced neurologists before an MRI scan was performed. Inclusion criteria for the PD group were patients diagnosed with clinically probable PD according to the 2015 Movement Disorder Society clinical diagnostic criteria for PD (Postuma et al., 2015); drug naïve (no prior anti-PD medications including levodopa, dopamine antagonists, catechol-*O*-methyl transferase inhibitors, or monoamine oxidase-B inhibitors); and Hoehn and Yahr Scale (H-Y) stage 1–2. Exclusion criteria for both patients and HCs were any primary neurological diseases (such as stroke, encephalitis, brain tumor), except PD; schizophrenia; bipolar disorder; substance abuse/dependence; and major white matter changes, infarction, or more than 10 lesions > 1 mm visible on structural MRI.

The Chinese version of Mini-Mental State Exam (MMSE), Montreal Cognitive Assessment (MoCA), Hamilton Anxiety Scale (HAMA), and Hamilton Depression Scale 24 (HAMD) were used to measure the general cognitive and psychomotor abilities of participants. PD-related motor symptoms were dependently evaluated by 2 experienced neurologists using the Chinese version of the motor section of the Unified Parkinson’s Disease Rating Scale (UPDRS III), and MRI was arranged before the dopaminergic therapy to avoid interference.

The study was approved by the Medical Ethics Committee of Tongji Medical College, Huazhong University of Science and Technology, and all participants provided written informed consent.

### Magnetic Resonance Imaging Acquisition and Magnetic Resonance Imaging Data Processing

Magnetic resonance imaging images were acquired using a 3.0 T MRI system (Ingenia 3.0T CX, Philips Healthcare, Best, Netherlands) equipped with a 32-channel head coil at the Union Hospital, Wuhan, Hubei, China. It was possible to fully communicate with all the participants; they were required to lay still in the supine position, and their ears were covered with headphones to block out the noise during scanning. Each participant was instructed to keep his/her mind relaxed, eyes closed, and awake during the MRI scan. After the MRI acquisition, a radiologist confirmed with each participant whether these instructions were followed. Anatomical images were acquired using a high-resolution T1-weighted 3-dimensional fast gradient echo sequence [“Turbo Field Echo,” Repetition Time (TR) = 11.2 s, Echo Time (TE) = 5.1 s, Flip Angle (FA) = 8°, acquired over a field of view of 384 × 384, slice thickness 0.7 mm, slices number = 258]. A multiband T2*-sensitive echo-planar imaging was used to collect rs-fMRI images (T2*-sensitive echo-planar imaging, TR/TE = 800/30 ms, multi-band SENSE acceleration factor = 6, 60 axial slices, slice thickness = 2.4 mm, matrix = 96 × 96, field of view = 200 × 200, 383 volumes for each participant) (Liu et al., 2022).

Functional images were preprocessed using the Data Processing and Analysis for Brain Imaging (DPABI) software toolbox^1^ (Yan et al., 2016). Preprocessing steps included slice-timing correction, motion correction, spatial normalization to the Montreal Neurological Institute (MNI) template, resampling into 3 × 3 × 3 mm^3^ size voxels, linear detrending, regressing out nuisance covariates (six head-motion parameters, cerebrospinal fluid, and white matter signals), low-pass filtering with a frequency cut-off of 0.01–0.08 Hz, and smoothing by 4 mm full width at half maximum (FWHM) Gaussian kernel.

### Functional Connectivity Analyses

Functional connectivity patterns of the brain networks were analyzed based on a prior Automated Anatomical Labeling (AAL) atlas (Tzourio-Mazoyer et al., 2002), which divides the brain into 90 (45 per hemisphere) cortical and subcortical regions of interest (ROIs), each representing a node of the network. The 90 AAL regions were clustered to 7 networks—the visual network (VSN), somatomotor network (SMN), dorsal attention network (DAN), ventral attention network (VAN), limbic network (LBN), frontoparietal network (FPN), and default mode network (DMN)—based on previous studies (Yeo et al., 2011; Long et al., 2020). Meanwhile, the bilateral caudate, putamen, pallidum, and thalamus were clustered as the deep gray matter network (DGN) (Baum et al., 2017; Long et al., 2020).

The first 10 volumes were removed because of signal equilibrium and participants’ adaptation to scanning noise. To represent the activity of a brain region, the mean time series for each region was first acquired by averaging the time series of all voxels within each AAL region [90 (ROI number) × 373 time points were obtained for each participant]. Pearson’s correlation coefficients (R-values) were calculated between the mean time series of each pair of regions (size: 90 × 90). These were then converted to nodal connectivity degree (η) using an exponential conversion method, while intra- and inter-network connections were calculated by averaging the correlation coefficients of all ROI pairs within or between each particular brain network, as previously described (Chen et al., 2016; Yang et al., 2016a; Long et al., 2020).

Functional connectivity analyses were performed at the integrity, network, and edge levels to study the shared general and distinct specific connectivity patterns between the PD and HC groups (Chen et al., 2016). The integrity level allows investigation of the information flow received from the brain in a specific node, while the network level includes intra- and inter-network analysis; intra-network analysis measures connectivity by averaging the transformed correlation coefficients within a network, while inter-network analysis measures the connectivity between 2 networks. At the edge level, we explored the functional connectivity between all possible node pairs. It is helpful to accurately study connectivity between nodes, as it may reveal the focal phenomena obscured by the integration of connected brain regions as networks (Brier et al., 2012).

### Statistical Analysis

An independent sample *t*-test was used to evaluate statistical significance was evaluated by (*p* < 0.05, corrected for multiple hypothesis testing through the permutation-based method at *p* < 0.05) of the Z scores at three levels between the two groups—after controlling for age, gender, and level of education —using a general linear model. Multiple regression analysis was performed to assess the correlation between altered functional connectivity metrics and MMSE, MoCA, HAMA, HAMD, and UPDRS-III scores of the PD patients; age, gender, and level of education were included as covariates (Peraza et al., 2017). The statistical significance threshold was set at *p* < 0.05 for exploratory purposes only.

## Results

### Clinical Findings

Fifty-six individuals with early-stage PD and 54 HCs were initially enrolled in the study. Six PD patients and four HCs were excluded because they refused to continue to participate in the study. Three PD patients were subsequently excluded owing to the presence of more than 10 cerebral lesions on MRI scan. Finally, 47 early-stage PD patients and 50 HCs completed the entire study and were included in the final analyses.

Demographic and clinical statistics are shown in Table 1. The mean age of the PD group was 62.34 ± 11.41 (mean ± SD) years, with a sex ratio of 18/29 (male/female). The mean age of the HC group was 61.32 ± 5.60 years, with a sex ratio of 19/31 (male/female). The mean educational years of PD patients was 9.83 ± 3.44, whereas that of HCs was 10.92 ± 3.18. No significant differences in age, sex, and educational years were found between the two groups. The mean disease duration of PD was 16.72 ± 12.08 months and mean H-Y stage was 1.60 ± 0.43. Scores related to MMSE (24.41 ± 3.75 vs. 25.58 ± 2.51, *p* = 0.04), MoCA (18.36 ± 6.21 vs. 23.56 ± 3.37, *p* < 0.001), HAMA (11.70 ± 7.48 vs. 4.68 ± 3.96, *p* < 0.001), and HAMD (11.40 ± 7.92 vs. 4.84 ± 3.25, *p* < 0.001) were significantly different between the PD and HC groups.

**TABLE 1:** Demographic and clinical characteristics of PD and HC group.

	PD (*n* = 47)	HC (*n* = 50)	*p*-value
Age, mean (SD)	62.34 (11.41)	61.32 (5.60)	0.582
Sex (Male/Female)	18/29	19/31	0.865
Educational years, mean (SD), years	9.82 (3.44)	10.92 (3.18)	0.109
Disease duration, mean (SD), months	16.72 (12.08)	–	–
H-Y Stage, mean (SD)	1.60 (0.43)	–	–
UPDRS III, mean (SD)	25.28 (12.03)	–	–
MMSE, mean (SD)	24.41 (3.75)	25.58 (2.51)	0.0481
MoCA, mean (SD)	18.36 (6.21)	23.56 (3.37)	<0.001
HAMA, mean (SD)	11.70 (7.48)	4.68 (3.96)	<0.001
HAMD, mean (SD)	11.40 (7.92)	4.84 (3.25)	<0.001

*PD, Parkinson’s disease; HC, healthy controls; H-Y Stage, Hoehn and Yahr stage; UPDRS III, motor section of the Unified Parkinson’s Disease Rating Scale; MMSE, Mini-Mental State Exam; MoCA, Montreal Cognitive Assessment; HAMA, Hamilton Anxiety Scale; HAMD, Hamilton Depression Scale.*

### Magnetic Resonance Imaging Findings

#### Integrity Level

**FIGURE 1:**
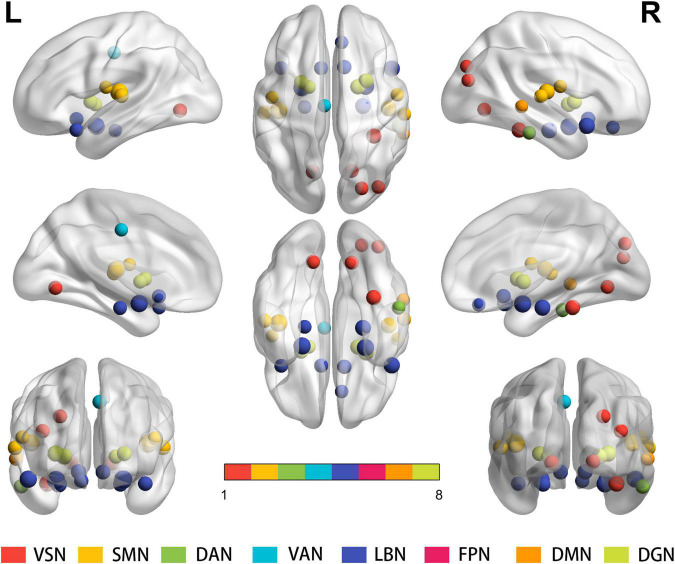
Results for the total degree of connectivity between nodes at the integrity level. Statistical analysis indicates significant between-group differences regarding the total functional connectivity at the 27 nodes distributed within the VSN, SMN, LBN and DGN (*P* < 0.05, FDR corrected). Same colored spheres represent ROIs from the same RSNs. ROIs, regions of interest; RSNs, resting-state network; VSN, the visual network; SMN, somatomotor network; DAN, dorsal attention network; VAN, ventral attention network; LBN, limbic network; FPN, frontoparietal network; DMN, default mode network; DGN, deep gray matter network; FDR, false discovery rate.

Statistical analysis revealed significant between-group differences in the degree of total functional connectivity at the 27 nodes distributed among the VSN, SMN, LBN, and DGN [*p* < 0.05, false discovery rate (FDR) corrected]. At the integrity level, significant differences were observed in the bilateral Rolandic operculum, bilateral olfactory cortex, right-side rectus, left media part of the cingulum, bilateral parahippocampal gyrus, bilateral amygdala, bilateral lingual gyrus, right side occipital and fusiform gyrus, bilateral putamen and pallidum, bilateral Heschl’s gyrus, bilateral superior temporal gyrus, bilateral superior temporal pole, right middle temporal gyrus, and inferior temporal gyrus between the two groups (Figure 1 and Supplementary Table 1).

#### Network Level

##### Alteration of Intra-Network Connections

**FIGURE 2:**
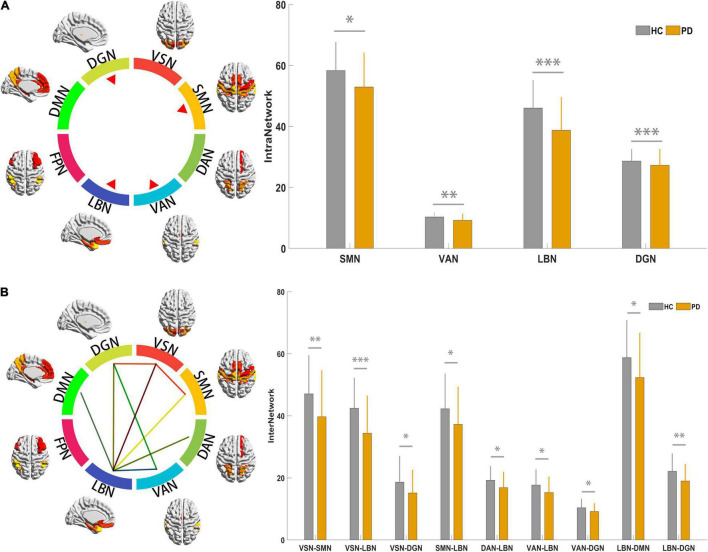
Results for connectivity among the 9 RSNs at the intra- and inter-network. **(A)** The colorful circle indicates the composite of 9 RSNs. The 4 red triangles indicate that the RSNs showed altered intra-network connections based on results indicating statistical significance. The histogram compares the 4 networks between PD and HC groups; the y-axis represents intra-network functional connectivity strength. **(B)** The colorful circle indicates the composite of 9 RSNs. The line linking 2 RSNs indicates altered individual inter-network connections based on statistically significant results. The histogram compares significantly decreased inter-network connections between the PD and HC groups; the y-axis represents the inter-network functional connectivity strength. Asterisks indicate significant group differences (*p < 0.05; ^**^p < 0.01, ^***^p < 0.001). PD, Parkinson’s disease; HC, healthy control; RSNs, resting-state network; VSN, the visual network; SMN, somatomotor network; DAN, dorsal attention network; VAN, ventral attention network; LBN, limbic network; FPN, frontoparietal network; DMN, default mode network; DGN, deep gray matter network.

Statistical analysis identified significantly decreased intra-network connections in the SMN, VAN, LBN, and DGN in PD patients; however, there were no significant intra-network connection differences in the VSN, DAN, FPN and DMN between the PD and HC groups (Figure 2A and Supplementary Table 2A).

##### Alteration of Inter-Network Connections

Statistical analysis identified significantly decreased inter-network connections between the VSN and SMN; VSN and DGN; LBN and VSN, SMN, DAN, VAN, DMN, and DGN; and VAN and DGN in PD patients (Figure 2B and Supplementary Table 2B).

#### Edge Level

**FIGURE 3:**
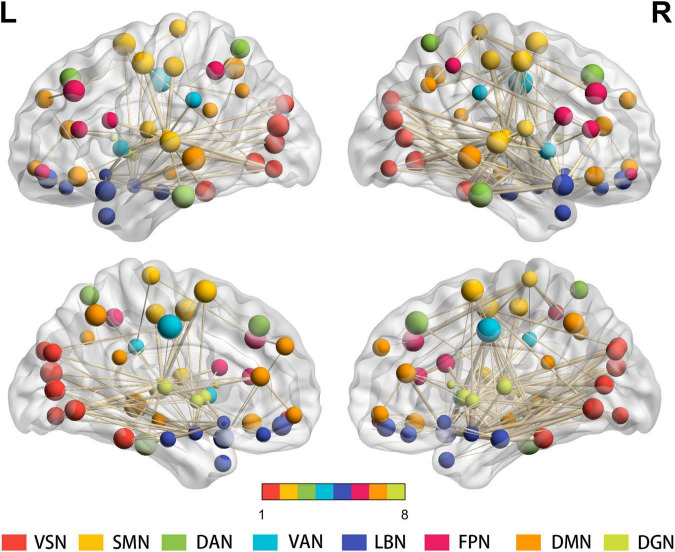
Results for the total degree of connectivity between nodes at the edge level. Four hundred forty-three widely distributed connectivity pairs—mainly regarding the intra-network connectivity of 8 RSNs, and inter-network connectivity between the SMN, DGN, and other RSNs—are shown (all *p* < 0.05, FDR corrected). The same color of spheres represented ROIs were from the same RSNs. PD, Parkinson’s disease; HC, healthy control; ROIs, regions of interest; RSNs, resting-state network; VSN, visual network; SMN, somatomotor network; DAN, dorsal attention network; VAN, ventral attention network; LBN, limbic network; FPN, frontoparietal network; DMN, default mode network; DGN, deep gray matter network; FDR, false discovery rate.

At the connectivity level, 443 altered intra- and inter-network functional connectivity pairs were identified between the two groups (all *p* < 0.05, FDR corrected) after controlling for age, sex, and education. Although widely distributed, these pairs mainly involved the intra-network connectivity of 8 resting-state networks (RSNs), and inter-network connectivity between the SMN, DGN, and other RSNs (Figure 3).

Additionally, results with global signal regression of three levels are shown in Supplementary Figure 2.

### Correlation Between Altered Connectivity and Clinical Information

**FIGURE 4:**
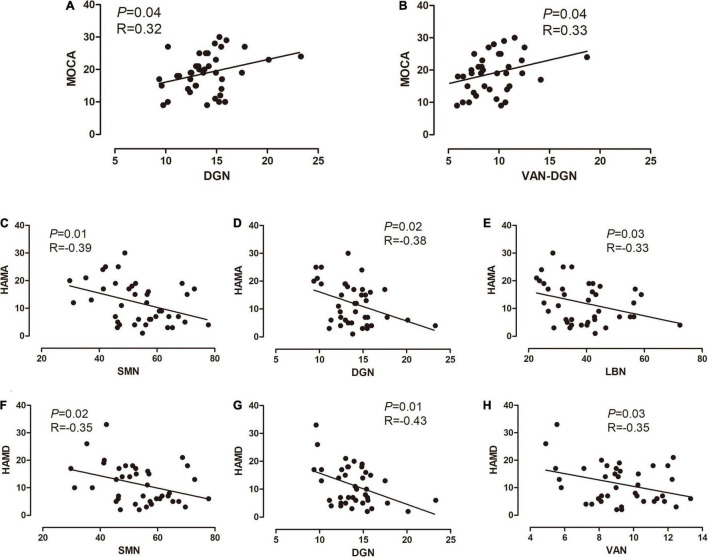
Correlations between altered connectivity and clinical information. **(A,B)** Correlations between the MoCA scores, intra-network connections of the DGN, and inter-network connections between the VAN and DGN, in the PD group. **(C–E)** Significant correlations between the HAMA scores and intra-network connections of the SMN, LBN, and DGN in the PD group. **(F–H)** Significant correlations between the HAMD scores, and intra-network connections of the SMN, VAN, and DGN. The x-axis represents the intra- or inter-network functional connectivity strength. PD, Parkinson’s disease; MoCA, Montreal Cognitive Assessment; SMN, somatomotor network; VAN ventral attention network; LBN, limbic network; DGN, deep gray matter network; HAMA, Hamilton Anxiety Scale; HAMD, Hamilton Depression Scale.

We identified significant correlations between MoCA scores and intra-network connections of the DGN, as well as between MoCA scores and inter-network connections between the VAN and DGN in the PD group. Additionally, significant correlations were found between the HAMA scores and intra-network connections of the SMN, LBN, and DGN, as well as between the HAMD scores and intra-network connections of the SMN, VAN, and DGN in in PD patients (Figure 4).

## Discussion

In our study, the brain was divided into 90 regions and eight networks, while the fMRI results of the two groups were analyzed at three different levels. We identified the first brain connections to be affected in the early stages of PD, providing a basis for our understanding of disease occurrence and development. Our results further supported the view that PD is a disconnection syndrome (Cronin-Golomb, 2010): functional disconnection among brain regions resulted in a series of clinical manifestations.

### Clinical Characteristics of Parkinson’s Disease

We included 47 drug naïve patients with PD to explore the correlation between the clinical characteristics of PD and brain connection. The mean H-Y stage of PD patients was 1.60 ± 0.43. Previous studies usually classified PD patients with H-Y stage 1–2 as early stage (Sung et al., 2010; Regnault et al., 2019; Hacker et al., 2020); therefore, we believed that the included patients were in the early stage of PD.

The mean UPDRS-III score of PD group was 25.283 ± 12.027. In previous studies of untreated PD patients, the mean *UPDRS-III* score ranged from 13.0 ± 7.4 to 25.9 ± 13.1 (Lee et al., 2015; Fründt et al., 2019; Hirata et al., 2020; White et al., 2020). Although the mean UPDRS-III score in this study was on the high side, it was not significantly higher than those reported in previous studies. This difference may be related to heterogeneity of PD.

The cognition of participants in the PD group was significantly lower than that in the control group, in particular the mean MoCA score of PD patients was 18.364 ± 6.210. This indicated that some patients in our study may have PD-related mild cognitive impairment (PD-MCI). At present, there is a lack of large-scale studies investigating prevalence of PD-MCI in early PD. A study involving 80 early PD patients reported that the prevalence of PD-MCI was 34% (Pfeiffer et al., 2014). In our study, the average MoCA score of PD patients was significantly lower than that of similar studies (Lee et al., 2015, 21917776; De Micco et al., 2021b). The MoCA score is related to age and educational years. A population-based study showed that the mean MoCA score of healthy individuals aged 60–70 years with less than 12 educational years was 19.30 ± 3.79 (Rossetti et al., 2011). Moreover, some words (such as “church” and “velvet”) adopted in the MoCA scale are unfamiliar to Chinese population, especially among the participants with poor education, which may contribute to the lower MoCA score. A meta-analysis revealed that PD-MCI was associated with a lower educational level (Baiano et al., 2020). In conclusion, it is reasonable to infer that the low MoCA score we obtained may be related to low educational attainment and cultural differences.

Anxiety and depression are the most common emotional disorders in PD patients. Although different scales are used for evaluation, they are closely related (Menza et al., 1993). HAMA and HAMD were used to evaluate anxiety and depression in this study, respectively. We found that the mean HAMA and HAMD scores were 11.70 ± 4.68 and 11.4 ± 7.92, respectively, which were significantly different from those of the HCs. This result indicated that patients included in our study had mild anxiety and depression. Emotional disorders can occur in the prodromal and early stage of PD. There is a lack of large-scale population-based studies on the prevalence of anxiety or depression in early PD. A study reported that the prevalence of depression in early PD was 27.6%(Ravina et al., 2007); however, the prevalence of anxiety in early PD is unknown. A review revealed that the prevalence of anxiety among all stage of PD patients ranges from 6.9 to 55%, indicating that prevalence may be affected by many factors. However, there are some difficulties in evaluating anxiety and depression in PD patients: symptoms often overlap with other non-motor symptoms of PD, and anti-PD drugs and PD-related cognitive impairment may also affect the results (Gallagher and Schrag, 2012). In this study, we included drug-naïve patients to exclude the effect of anti-PD drugs and more accurately study the brain connectivity of depressive and anxiety symptoms in early PD patients.

### Alteration of the Somatomotor Network

During the process from command generation to execution, the sensory network modifies the command generated by the primary motor area, ensuring its correct execution (Makino et al., 2016); abnormalities in the sensory motor network may therefore be a basis for PD motor symptoms. Our study showed that Heschl’s gyrus, superior temporal gyrus, and Rolandic operculum had abnormal connections with other brain regions; inter-network analysis also confirmed that PD patients had abnormal connections between the SMN and other networks, consistent with the findings of previous studies (Koshimori et al., 2016; Huang et al., 2017; Helmich, 2018). Motor neurons in the anterior central gyrus are the main origin of nerve fibers responsible for fine and purposeful single movements. Notably, edge-level analysis indicated a decrease in connections between the anterior central gyrus and multiple brain regions of the VSN and LBN, including the amygdala, parahippocampal gyrus, lingual gyrus, and superior occipital gyrus.

Abnormal connections between the anterior central gyrus and these brain regions indicate that regulation of limb movement is impaired in early PD; after brain network dysfunction, this will be compensated for by establishing new brain connections. Increased abnormal brain connections were found in the motor-related brain regions in PD; we speculate that they were produced early in the disease course to compensate for damaged brain connections. These compensatory brain connections only appeared in specific brain regions and were concentrated in the connections between the paracentral lobule and other brain regions. It is plausible that compensatory brain connections are produced in the preclinical stage of PD.

### Alteration of the Deep Gray Matter Network

The DGN, composed of deep gray matter nuclei, plays an important role in many aspects, including learning, memory, action execution, and somatosensory movement. Many motor disorders are related to deep gray matter lesions. Our results showed abnormal connections within the DGN in PD patients, reflecting a decrease in connections between the putamen and pallidum areas.

Globus pallidus stimulation can be used to improve the brain connectivity of DGN in order to treat advanced PD (Tsuboi et al., 2021). Integrity level analysis showed that the connections among the putamen, pallidum, and other parts of the brain were significantly reduced in PD patients; this is consistent with the clinical manifestation of bradykinesia caused by dopamine metabolism dysfunction in the early stages of PD. Previous studies have shown that this connectivity disorder is also related to the language dysfunction in early PD (Manes et al., 2018).

### Alteration of the Limbic Network

The LBN can be subdivided into four parts: the hippocampal-diencephalic, parahippocampal-retrosplenial, temporo-amygdala-orbitofrontal, and dorsomedial default-mode networks (Catani et al., 2013). The LBN has complex functions, including cognitive memory, emotional control, tactile feedback, and movement. There are only a few studies on the relationship between LBN abnormalities and clinical features in PD; currently, fatigue, apathy, depression, hyposmia, and freezing of gait are considered to be related to lesions in the limbic system (Remy et al., 2005; Su et al., 2015; Gilat et al., 2018; Prange et al., 2019).

Widespread anomalies were found in early PD edge network connections. Integrity level analysis revealed abnormal connections between multiple brain regions in the temporo-amygdala-orbitofrontal network in early PD, associating with the integration of emotional states and behavior. This may be related to the early appearance of cognitive impairment, anxiety, and depression in some PD patients. The olfactory center is also located in this network; hence, abnormal connectivity can explain the performance of olfactory impairment and loss in PD patients. The hippocampal-diencephalic and parahippocampal-retrosplenial networks are mainly related to memory and spatial orientation (Aggleton, 2008; Vann et al., 2009). In PD patients, connections between the parahippocampal gyrus and other brain regions are reduced, which may also cause cognitive impairment.

Widely abnormal connections demonstrated by network level analysis between the LBN and other networks, indicate that the LBN plays an important role in the entire brain network. Although the LBN participates in the normal physiological function of other brain networks, this also suggests that LBN lesions can cause multiple network dysfunctions in PD, which may relate to a variety of non-motor preclinical and early-stage PD symptoms. In addition to non-motor symptoms interacting with each other (Engels et al., 2019), the wide range of abnormal connections in the LBN may be a bridge linking motor and non-motor symptoms.

Edge-level analysis showed that extensive connectivity abnormalities were concentrated in the connections among the amygdala, parahippocampal gyrus, and other brain networks. Amygdala dysfunction has been associated with PD depression (Huang et al., 2015); additionally, there is a wide, two-way connection between the amygdala and other brain networks. We found that this extensive connection was also damaged; therefore, amygdala dysfunction may also be involved in PD related symptoms, excluding depression. The central amygdaloid nucleus (CeA) contains many dopamine fiber terminals, with dopamine being one of the most important neurotransmitters (Hasue and Shammah-Lagnado, 2002). In early PD, approximately 80% of striatal dopamine and 50% of substantia nigra neurons are lost (Samii et al., 2004), suggesting that CeA function is also significantly impaired; this neuron loss may be the anatomical basis of a series of PD symptoms. The parahippocampal gyrus—an important afferent source of the hippocampus—is the starting site of the Papez circuit and participates in high-level neural activities such as emotion, learning, and memory (Choi et al., 2019).

### Alteration of the Visual Network

Following the increased attention to non-motor symptoms of PD, the visual system changes in PD, including pathological changes, such as α-synuclein deposition, and retinal dopamine deficiency, have been recently studied (Guo et al., 2018). The prolonged latency of visual evoked potential suggests that the lesions involve the visual electrophysiological pathway in PD (He et al., 2018). Hallucination is a common non-motor symptom during middle-stage and late-stage PD; however, other visual symptoms, such as dyslexia (Archibald et al., 2011; Urwyler et al., 2014), often appear in the early stage.

All participants with early PD did not report hallucinations; however, our results revealed significantly abnormal VSN connectivity of PD patients. Affected networks were closely related to the motor and non-motor symptoms of PD, suggesting that they affect the visual network, thus becoming the pathological basis of visual symptoms. Early PD affects the connections between the VSN and other networks. The reduced brain connections may weaken the ability to regulate visual sensation and movement, which may be involved in the occurrence of some PD symptoms.

### Correlations Between Altered Connectivity and Clinical Information

Although previous studies found an association between the UPDRS III score and brain connectivity (Day et al., 2019), we did not observe similar results. The participants in our study had early-stage PD; thus, the degree of brain connectivity damage may not have matched the clinical symptoms. Additionally, the absence of advanced PD patients was not conducive to regression analysis.

We found a linear relationship between the MoCA score and the strength of both DGN and DGN-VAN connectivity. Previous studies suggested that the VAN is related to hallucination in PD (Shine et al., 2011; Sawczak et al., 2019), which was not reported in relation to cognitive impairment in PD. Improving the function of the DGN and DGN-VAN may contribute to the treatment of PD-related cognitive impairment; however, this finding should be validated in longitudinal studies. Additionally, this linear relationship was not found in the MMSE score, as the MoCA may be more sensitive and discriminative to the screening of cognitive impairment.

Depression and anxiety are common non-motor symptoms in early PD. A disorder in the dopamine and serotonin levels is considered to be involved (Thobois et al., 2017); however, the pathogenesis is unclear. By analyzing the correlation between HAMA/HAMD scores and brain connection at the network level, we found a linear relationship between the HAMA score and the function connectivity strength of the SMN, DGN, and LBN; a similar result was obtained regarding the function connectivity strength of the SMN, DGN, and VAN. Previous studies have reached similar conclusions (Hu et al., 2015; Liao et al., 2020; Qiu et al., 2021), and our findings provide guidance for further studies investigating early PD pathogenesis in drug-naïve patients.

### Limitations

Some shortcomings and limitations of the present study should be mentioned. First, this was a cross-sectional study. Although the latest diagnostic criteria for PD were adopted, limited by the misdiagnosis rate of early-stage PD was high, some Parkinson’s syndrome patients might be included in this study, thus potentially affecting the results. In the future, participants should be followed up to dynamically observe the development of the disease and changes in brain connectivity. Second, considering the unstable image signal of the cerebellum, we did not assess the changes in cerebellar functional connections; a previous study revealed changes in cerebellar functional connections within the cerebellum, and between the cerebellum and cerebral hemispheres in early PD, suggesting that the cerebellum plays a primarily compensatory role in early PD (Xu et al., 2019). It is important to note that multiple regression analyses were used for exploratory analysis in the study of correlation between brain connectivity and clinical scales. But multiple comparison correction was not performed, which may lead to false positive results and so more research is needed. Additionally, geographical factors of participants may have caused bias as this was a single-center study.

## Conclusion

**FIGURE 5:**
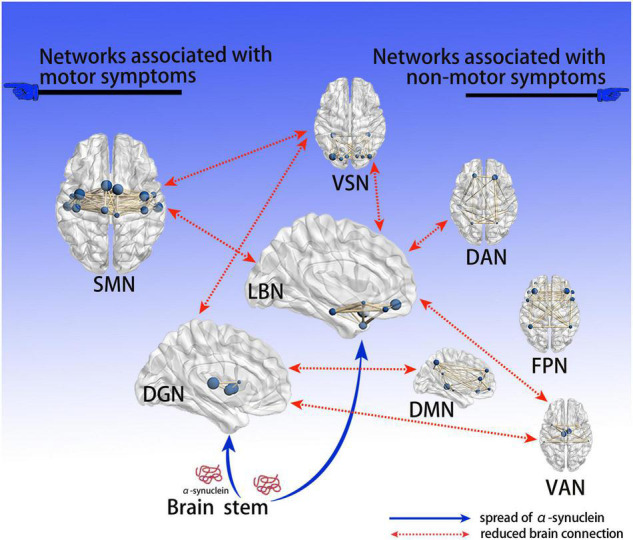
Hypothetical model explaining how deposition of α-syn affects brain networks. Based on Braak’s PD α-syn propagation theory, since the deep gray matter and limbic systems are close to the brain stem, they are the first to be affected by α-syn; the abnormal brain network connections found are consistent with the propagation path of α-syn. Neurotoxic α-syn deposits in these 2 encephalic regions, affecting the function of neurons; the connection of related brain networks is then affected, resulting in a series of clinical PD symptoms. We believe that dysfunctional brain connections of the DGN and SMN are associated with motor symptoms, while the DMN, DAN, FPN, and VAN are associated with non-motor symptoms; notably, the LBN and VSN are associated with both motor and non-motor symptoms. PD, Parkinson’s disease; α-syn, α-synuclein; VSN, visual network; SMN, somatomotor network; DAN, dorsal attention network; VAN, ventral attention network; LBN, limbic network; FPN, frontoparietal network; DMN, default mode network; DGN, deep gray matter network.

To our knowledge, our study is the first to demonstrate variations in whole-brain connections at three different levels in drug-naïve patients with early PD. We revealed that major changes occurred in the SMN, DGN, LBN, and VSN, which may be relevant to both motor and non-motor symptoms in drug-naïve early PD patients included in our study. Our results further supported the view that PD is a disconnection syndrome. Based on Braak’s PD hypothesis (Braak et al., 2004) and our findings, we proposed a hypothetical model to explain how the deposition of α-synuclein affects brain networks (Figure 5). Among the altered networks, we conjectured that the LBN was closely related to non-motor symptoms; thus, this warrants further investigation.

## Data Availability Statement

The original contributions presented in the study are included in the article/Supplementary Material, further inquiries can be directed to the corresponding author/s.

## Ethics Statement

The studies involving human participants were reviewed and approved by Medical Ethics Committee of Tongji Medical College, Huazhong University of Science and Technology. The patients/participants provided their written informed consent to participate in this study.

## Author Contributions

WZ, WF, XC, and YaX designed the study. WZ, XL, LL, ZC, XZ, XY, CC, YW, YuX, XC, and YaX conducted participant enrollment and evaluation. WF, XK, and XL implemented MRI scanning. WZ and WF wrote the original draft. XC and YaX supervised the study. WF, XC, and YaX contributed to funding acquisition. All authors reviewed and edited the manuscript.

## Conflict of Interest

The authors declare that the research was conducted in the absence of any commercial or financial relationships that could be construed as a potential conflict of interest.

## Publisher’s Note

All claims expressed in this article are solely those of the authors and do not necessarily represent those of their affiliated organizations, or those of the publisher, the editors and the reviewers. Any product that may be evaluated in this article, or claim that may be made by its manufacturer, is not guaranteed or endorsed by the publisher.

## Footnotes

1
http://rfmri.org/dpabi

## Supplementary Material

Supplementary Figure 1 Flow diagram of the study. PD, Parkinson’s disease; HC, healthy control; MRI, magnetic resonance imaging; UPDRS III, motor section of the Unified Parkinson’s Disease Rating Scale; MMSE, Mini-Mental State Exam; MoCA, Montreal Cognitive Assessment; HAMA, Hamilton Anxiety Scale; HAMD, Hamilton Depression Scale.

Supplementary Figure 2 Results of functional connectivity analyses at 3 levels with global signal regression. **(A)** Statistical analysis indicates significant between-group differences regarding the total functional connectivity at the 7 nodes (*P* < 0.05, FDR corrected). **(B)** The colorful circle indicates the composite of 9 RSNs. The 4 red triangles indicate that the RSNs showed altered intra-network connections based on results indicating statistical significance. The histogram compares the 4 networks between PD and HC groups; the y-axis represents intra-network functional connectivity strength. **(C)** The colorful circle indicates the composite of 9 RSNs. The line linking 2 RSNs indicates altered individual inter-network connections based on statistically significant results. The histogram compares significantly decreased inter-network connections between the PD and HC groups; the y-axis represents the inter-network functional connectivity strength. **(D)** Two hundred twenty-one widely distributed connectivity pairs are shown (all *p* < 0.05, FDR corrected). The same color of spheres represented ROIs were from the same RSNs. Asterisks indicate significant group differences (**p* < 0.05, ^**^*p* < 0.01, ^***^*p* < 0.001). PD, Parkinson’s disease; HC, healthy control; ROIs, regions of interest; RSNs, resting-state network; VSN, visual network; SMN, somatomotor network; DAN, dorsal attention network; VAN, ventral attention network; LBN, limbic network; FPN, frontoparietal network; DMN, default mode network; DGN, deep gray matter network; FDR, false discovery rate.
