# Supplementary figures and images for: Altered Intra- and Inter-Network Connectivity in Drug-Naïve Patients With Early Parkinson’s Disease
Source: Front Aging Neurosci. 2022 Feb 14;14:783634. doi: 10.3389/fnagi.2022.783634 (PMC8884479; doi:10.3389/fnagi.2022.783634)

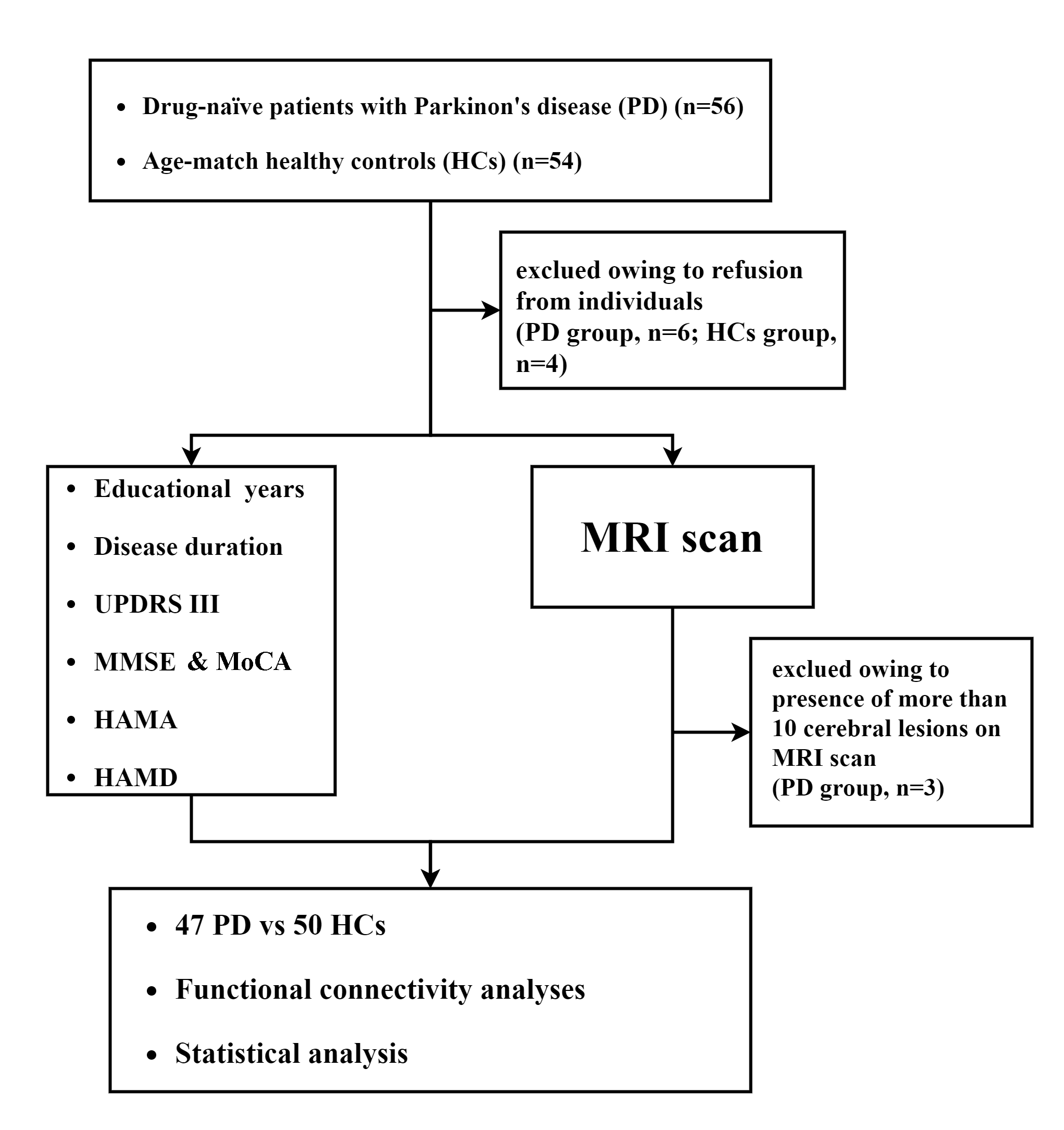

Supplement: Supplementary Figure 1 — Flow diagram of the study. PD, Parkinson’s disease; HC, healthy control; MRI, magnetic resonance imaging; UPDRS III, motor section of the Unified Parkinson’s Disease Rating Scale; MMSE, Mini-Mental State Exam; MoCA, Montreal Cognitive Assessment; HAMA, Hamilton Anxiety Scale; HAMD, Hamilton Depression Scale. [file Image_1.tif]

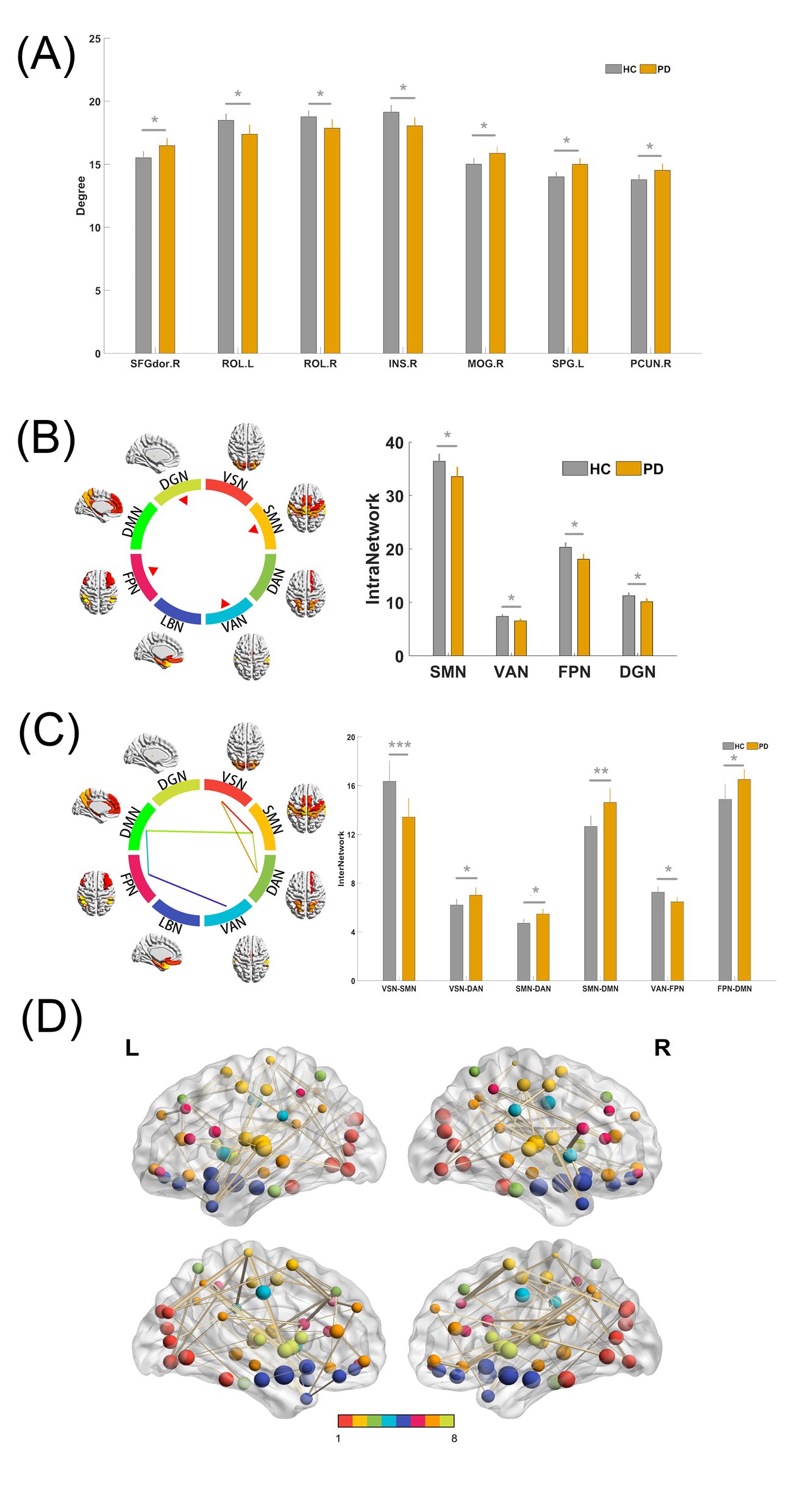

Supplement: Supplementary Figure 2 — Results of functional connectivity analyses at 3 levels with global signal regression. (A) Statistical analysis indicates significant between-group differences regarding the total functional connectivity at the 7 nodes (P < 0.05, FDR corrected). (B) The colorful circle indicates the composite of 9 RSNs. The 4 red triangles indicate that the RSNs showed altered intra-network connections based on results indicating statistical significance. The histogram compares the 4 networks between PD and HC groups; the y-axis represents intra-network functional connectivity strength. (C) The colorful circle indicates the composite of 9 RSNs. The line linking 2 RSNs indicates altered individual inter-network connections based on statistically significant results. The histogram compares significantly decreased inter-network connections between the PD and HC groups; the y-axis represents the inter-network functional connectivity strength. (D) Two hundred twenty-one widely distributed connectivity pairs are shown (all p < 0.05, FDR corrected). The same color of spheres represented ROIs were from the same RSNs. Asterisks indicate significant group differences (*p < 0.05, **p < 0.01, ***p < 0.001). PD, Parkinson’s disease; HC, healthy control; ROIs, regions of interest; RSNs, resting-state network; VSN, visual network; SMN, somatomotor network; DAN, dorsal attention network; VAN, ventral attention network; LBN, limbic network; FPN, frontoparietal network; DMN, default mode network; DGN, deep gray matter network; FDR, false discovery rate. [file Image_2.TIF]
