# Supplementary material for: Altered Intra- and Inter-Network Connectivity in Drug-Naïve Patients With Early Parkinson’s Disease
Source: Front Aging Neurosci. 2022 Feb 14;14:783634. doi: 10.3389/fnagi.2022.783634 (PMC8884479; doi:10.3389/fnagi.2022.783634)
Supplement: Supplementary file 3 [file Table_1.docx]

# Supplemental material

**Supplementary Table 1. Significant group differences at the integrity level**

| AAL regions | Yeo's network index | HC Degree, mean (SD) | PD Degree, mean (SD) | t value | p value |
| --- | --- | --- | --- | --- | --- |
| Lingual_L | Visual | 43.4 (9.73) | 37.92 (12.07) | 2.4807 | 0.0149 |
| Lingual_R | Visual | 43.64 (10.19) | 38.54 (11.56) | 2.3188 | 0.0225 |
| Occipital_Sup_R | Visual | 40.1 (9.47) | 35.62 (11.01) | 2.1621 | 0.0331 |
| Occipital_Mid_R | Visual | 41.88 (8.99) | 37.39 (11.79) | 2.1238 | 0.0363 |
| Fusiform_R | Visual | 43.24 (9.98) | 36.99 (10.83) | 2.9745 | 0.0037 |
| Rolandic_Oper_L | Somatomotor | 39.39 (10.58) | 34.93 (11.35) | 2.0088 | 0.0474 |
| Rolandic_Oper_R | Somatomotor | 39.28 (11.03) | 34.61 (11.36) | 2.0623 | 0.0419 |
| Heschl_L | Somatomotor | 37.95 (11.48) | 28.3 (11.91) | 4.0840 | 0.0001 |
| Heschl_R | Somatomotor | 37.83 (12.44) | 29.76 (11.4) | 3.3444 | 0.0012 |
| Temporal_Sup_L | Somatomotor | 44.11 (10.7) | 37.96 (12.14) | 2.6628 | 0.0091 |
| Temporal_Sup_R | Somatomotor | 44.78 (10.58) | 39.53 (12.19) | 2.2800 | 0.0248 |
| Temporal_Inf_R | Dorsal Attention | 47.48 (9.48) | 42.94 (10.68) | 2.2259 | 0.0284 |
| Cingulum_Mid_L | Ventral Attention | 47.33 (9.87) | 42.9 (11.18) | 2.0820 | 0.0400 |
| Olfactory_L | Limbic | 35.04 (9.46) | 30.06 (10.75) | 2.4336 | 0.0168 |
| Olfactory_R | Limbic | 36.51 (11.44) | 30.72 (10.73) | 2.5814 | 0.0114 |
| Rectus_R | Limbic | 38.31 (10.5) | 33.56 (11.24) | 2.1628 | 0.0330 |
| ParaHippocampal_L | Limbic | 38.1 (11.43) | 32.96 (10.8) | 2.2827 | 0.0247 |
| ParaHippocampal_R | Limbic | 42.54 (10.8) | 35.21 (11.09) | 3.3163 | 0.0013 |
| Amygdala_L | Limbic | 38.43 (9.81) | 29.51 (12.28) | 3.9806 | 0.0001 |
| Amygdala_R | Limbic | 39.64 (10.01) | 31.14 (11.22) | 3.9610 | 0.0001 |
| Temporal_Pole_Sup_L | Limbic | 43.34 (10.65) | 37.93 (12.8) | 2.2762 | 0.0251 |
| Temporal_Pole_Sup_R | Limbic | 45.64 (10.55) | 37.4 (12.67) | 3.5023 | 0.0007 |
| Temporal_Mid_R | Default Mode | 45.95 (9.8) | 41.61 (11.56) | 2.0048 | 0.0478 |
| Putamen_L | Deep Grey Matter | 37.27 (9.91) | 32.45 (9.39) | 2.4660 | 0.0154 |
| Putamen_R | Deep Grey Matter | 38.67 (10.02) | 32.68 (10.03) | 2.9538 | 0.0039 |
| Pallidum_L | Deep Grey Matter | 35.5 (10.04) | 31.18 (9.58) | 2.1807 | 0.0316 |
| Pallidum_R | Deep Grey Matter | 38.11 (10.91) | 31.54 (10.91) | 2.9788 | 0.0037 |

AAL regions refers to Tzourio-Mazoyer N, Landeau B, Papathanassiou D, et al. Automated anatomical labeling of activations in SPM using a macroscopic anatomical parcellation of the MNI MRI single-subject brain. Neuroimage. 2002;15(1):273-289. doi:10.1006/nimg.2001.0978

Abbreviations: AAL, Automated Anatomical Labeling; PD=Parkinson’s disease; HC, healthy controls;

**Supplementary Table 2A. Group differences at the intranetwork level**

| Yeo's network index | | HC Degree, mean (SD) | PD Degree, mean (SD) | t value | p value |
| --- | --- | --- | --- | --- | --- |
| Visual | 65.33 (9.11) | | 64.08 (10.47) | -0.6355 | 0.5266 |
| Somatomotor | 58.33 (9.43) | | 52.91 (11.19) | -2.5962 | 0.0109 |
| Dorsal Attention | 9.46 (1.9) | | 9.31 (1.97) | -0.3756 | 0.708 |
| Ventral Attention | 10.28 (1.5) | | 9.17 (2.19) | -2.9446 | 0.0041 |
| Limbic | 46.01 (9.15) | | 38.72 (10.89) | -3.5914 | 0.0005 |
| Frontoparietal | 28.63 (3.98) | | 27.25 (5.31) | -1.4548 | 0.149 |
| Default Mode | 80.97 (14.67) | | 79.7 (17.6) | -0.3888 | 0.6982 |
| Deep Grey Matter | 15.94 (2.52) | | 13.84 (2.9) | -3.8439 | 0.0002 |

Abbreviations: PD,Parkinson’s disease; HC, healthy controls;

**Supplementary Table 2B. Significant group differences at the internetwork level**

| Yeo's network index | HC Degree, mean (SD) | | | PD Degree, mean (SD) | t value | | p value |
| --- | --- | --- | --- | --- | --- | --- | --- |
| VSN-SMN | | 47.08 (12.44) | 39.71 (14.97) | | | -2.6513 | 0.0094 |
| VSN-LBN | | 42.42 (9.87) | 34.39 (12.13) | | | -3.6006 | 0.0005 |
| VSN-DGN | | 18.62 (8.42) | 15.13 (7.48) | | | -2.1653 | 0.0328 |
| SMN-LBN | | 42.28 (11.33) | 37.23 (12.19) | | | -2.1261 | 0.0361 |
| DAN-LBN | | 19.23 (4.58) | 16.86 (5.09) | | | -2.4299 | 0.017 |
| VAN-LBN | | 17.68 (5.11) | 15.27 (5.16) | | | -2.3224 | 0.0223 |
| VAN-DGN | | 10.37 (2.97) | 9.17 (2.67) | | | -2.1013 | 0.0382 |
| LBN-DMN | | 58.76 (12.08) | 52.34 (14.38) | | | -2.3978 | 0.0184 |
| LBN-DGN | | 22.14 (5.78) | 19 (5.48) | | | -2.7608 | 0.0069 |

Abbreviations: PD, Parkinson’s disease; HC, healthy controls; VSN, the visual networks; SMN, somatomotor networks; DAN, dorsal attention networks; VAN, ventral attention networks; LBN, limbic networks; DMN, default mode networks; DGN, deep gray matter network
